# A large population‐based study on the prevalence of electrocardiographic abnormalities: A result of Mashhad stroke and heart atherosclerotic disorder cohort study

**DOI:** 10.1111/anec.13086

**Published:** 2023-09-03

**Authors:** Sara Saffar Soflaei, Mahmoud Ebrahimi, Hamid Reza Rahimi, AmirAli Moodi Ghalibaf, Maryam Jafari, Hedieh Alimi, Nasrin Talkhi, Bahram Shahri, Alireza Heidari‐Bakavoli, Fatemeh Malakouti, Mahla Velayati, Reza Assaran‐Darban, Malihehsadat Abedsaeidi, Farnoosh Azarian, MohammadReza Latifi, Mohammad Reza Mohammad Taghizadeh Sarabi, Gordon A. Ferns, Habibollah Esmaily, Mohsen Moohebati, Majid Ghayour‐Mobarhan

**Affiliations:** ^1^ International UNESCO Center for Health‐Related Basic Sciences and Human Nutrition Mashhad University of Medical Sciences Mashhad Iran; ^2^ Vascular and Endovascular Research Center, Faculty of Medicine Mashhad University of Medical Sciences Mashhad Iran; ^3^ Applied Biomedical Research Center Mashhad University of Medical Sciences Mashhad Iran; ^4^ Student Research Committee, Faculty of Medicine Birjand University of Medical Sciences Birjand Iran; ^5^ Student Research Committee, Anzali International Medical Campus Guilan University of Medical Sciences Guilan Iran; ^6^ Department of Biostatistics, School of Health Mashhad University of Medical Sciences Mashhad Iran; ^7^ Department of Cardiology, Faculty of Medicine Mashhad University of Medical Sciences Mashhad Iran; ^8^ Student Research Committee, Faculty of Medicine Mashhad University of Medical Sciences Mashhad Iran; ^9^ Department of Biology, Mashhad Branch Islamic Azad University Mashhad Iran; ^10^ Division of Medical Education Brighton and Sussex Medical School Brighton UK; ^11^ Social Determinants of Health Research Center Mashhad University of Medical Sciences Mashhad Iran; ^12^ Metabolic Syndrome Research Center, Faculty of Medicine Mashhad University of Medical Sciences Mashhad Iran

**Keywords:** arrhythmia, electrocardiogram abnormalities, prevalence, Q wave, ST depression Minnesota code, T wave

## Abstract

**Background:**

Twelve‐lead electrocardiogram (ECG) is a common and inexpensive tool for the diagnostic workup of patients with suspected cardiovascular disease, both in clinical and epidemiological settings. The present study was designed to evaluate ECG abnormalities in Mashhad population.

**Methods:**

ECGs were taken as part of MASHAD cohort study (phase1) and were coded according to the Minnesota coding criteria. Data were analyzed using SPSS.

**Results:**

Total 9035 ECGs were available for final analysis including 3615 (40.0%) male and 5420 (60.0%) female. Among ECG abnormalities precordial Q wave, major T‐wave abnormalities, inferior Q wave, sinus bradycardia, and left axis deviation were the most prevalent abnormalities. The frequency of precordial and inferior Q wave, inferior QS pattern, major and minor ST abnormalities, major and minor T abnormalities, Wolff‐Parkinson‐White and Brugada pattern, sinus bradycardia, sinus tachycardia, left axis deviation, ST elevation, and tall T wave were significantly different between two genders. Moreover, the frequency of Q wave in precordial and aVL leads, QS pattern in precordial and inferior leads, major and minor T‐wave abnormalities, Wolff‐Parkinson‐White, atrial fibrillation, sinus bradycardia, left axis deviation, and ST elevation were significantly different in different age groups. A comparison of the heart rate, P‐wave duration, and QRS duration between men and women indicated that there was a significant difference.

**Conclusions:**

Our finding indicated that the prevalence ECG abnormalities are different between men and women and also it varied in different age groups.

## Graphical abstract

Prevalence of ECG abnormalities defined by Minnesota coding system in men and women using word cloud graph. Among ECG abnormalities precordial Q wave, major T wave abnormalities, inferior Q wave, sinus bradycardia and left axis deviation were the most prevalent abnormalities. Our finding indicated that the prevalence ECG abnormalities are different between men and women and also it varied in different age groups.

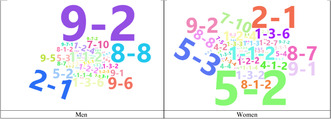

## 1 INTRODUCTION

Cardiovascular diseases (CVDs) are one of the major causes of morbidity and mortality in both developing or developed countries (Amini et al., 2021). Recent global studies indicate that CVDs affects more than half a billion people (Roth et al., 2020). The World Health Organization reports that CVDs are causing approximately 17.9 million lives each year; however, 80% of these global deaths occur in low‐ and middle‐income countries where CVDs and their risk factors are increasing as a result of a steady epidemiologic transition (Şahin & İlgün, 2022). Despite many endogenous, exogenous, environmental, genetic, and epigenetic factors that can increase the risk of CVD incidence, an early identification of CVDs in both individuals and communities is important in order to prevent exposure to its risk factors and reduce CVD‐related mortality (Alzahrani et al., 2019; Francula‐Zaninovic & Nola, 2018; Kakadiaris et al., 2018). Therefore, having low‐cost, mobile, available, and reliable equipment to detect cardiovascular events can make us capable of managing CVDs as well as possible (Bakirhan et al., 2018).

The introduction of the electrocardiogram (ECG) made an important impact on diagnostic medicine, especially in cardiovascular disease detection (Javaid et al., 2022). Today, 12‐lead ECG has become a common, important, routine, repeatable, and inexpensive tool for the diagnostic workup of cardiovascular disease, both in clinical and epidemiological settings (Maron et al., 2014). One important barrier to its use was the lack of precise criteria for ECG interpretation in large epidemiological studies, the development of the Minnesota coding system allowed to categorize and compare ECG abnormalities with each other (Prineas et al., 2009). On the contrary, to have an accurate and reliable view of cardiovascular events, ECG abnormalities, and the Minnesota coding system, large population‐based studies are required. A few studies in Poland and China showed that gender and age were significantly associated with ECG abnormalities; however, the frequency of the ECG abnormalities differed in each population with another (Piwońska et al., 2019; Yu et al., 2020).

The impact of CVD events on human health and lives makes it important to recognize these events at an early stage. There are few studies on general populations that have evaluate abnormal findings of resting ECG; therefore, the present study was designed to evaluate the frequency and type of abnormal findings in resting ECGs of Mashhad stroke and heart atherosclerotic disorder (MASHAD) study participants.

## 2 METHODS

### 2.1 Study design and participants

This cross‐sectional study was carried out on the population‐based (MASHAD) study that was started in 2010 (Ghayour‐Mobarhan et al., 2015). According to the MASHAD study protocol, participants were recruited from three urban regions in Mashhad, the second big city of Iran, using a stratified cluster random sampling method. A total of 9704 aged between 35 and 65 were included in the study (Ghayour‐Mobarhan et al., 2015). Demographic, anthropometric, physical exercise, smoking, nutritional intakes, and disease history of the subjects were recorded. Fasting blood was obtained from all participants, and fasting blood glucose (FBG) and lipid profile were measured with routine methods. Diabetes mellitus (DM) was defined as FBG ≥126 mg/dL or history of DM. Hypertension (HTN) was defined as systolic blood pressure ≥140 mmHg or diastolic blood pressure ≥90 mmHg or history of HTN. CVD was confirmed by a cardiologist according to CVD risk‐related questionnaire and a resting 12‐lead ECG and complementary test if needed. All participants in the MASHAD study gave informed consent prior to data collection for their anonymized clinical data to be recorded, and all procedures of the present study were approved by Mashhad university of Medical Sciences, Mashhad, Iran.

### 2.2 Resting 12‐lead ECG and Minnesota coding system

A single resting 12‐lead ECG was obtained from each participant on paper. ECGs were scanned and archived digitally. Only Standard ECGs with the speed of 25 mm/s and voltage of 10 mm/mV including 9035 ECGs were coded according to Minnesota (MN) coding system (Prineas et al., 2009). Ten medical students were trained to generate the codes with the aid of a designated digital platform according to Minnesota manual (Prineas et al., 2009). Average inter‐observer variabilities for quantitative variables were 10%, while intra‐observer variability for these variables was 5.3%. Inter‐observer and intra‐observer agreement for MN codes were 0.97 and 0.99, respectively. Total 5% of all ECGs was recorded by three cardiologists. Total 94% of generated codes by the trained staff were compatible with the codes generated by cardiologists.

Min and Max QT was corrected according to Bazett's formula (QTc = QT/RR^1/2^).

### 2.3 Statistical analysis

Data were analyzed using SPSS software version 21. Results are expressed as mean ± standard deviation, absolute numbers (*n*), and frequencies (%). The chi‐squared test was used to compare ECG abnormalities in gender and three age groups. A *p* < .05 was considered statistically significant.

## 3 RESULTS

Total 9035 available and readable ECGs were entered into the final analysis including 3615 (40.0%) men and 5420 (60.0%) women. Mean ± SD age of the participants was 48.91 ± 8.39 in men and 47.60 ± 8.09 in women. Men and women were categorized in three age groups including 35–44 years (*N* = 1339 in men and *N* = 2256 in women), 45–54 years (*N* = 1473 in men and *N* = 2287 in women), and 55–65 years (*N* = 1022 in men and *N* = 1189 in women). The data revealed a higher prevalence of either current or ex‐smoking and history of CVDs in males than females (41.9% vs. 23.9%, and 2.5% vs. 1.1%). Baseline characteristics including employment status, smoking status, BMI, DM, HTN, and CVD are indicated in Table 1.

**TABLE 1:** Baseline characteristics of studied population according to gender and age groups.

Baseline characteristics	Men				Women			
	Total	35–44	45–54	55–65	Total	35–44	45–54	55–65
Employment status								
Student	10 (0.3)	5 (0.4)	3 (0.2)	2 (0.2)	8 (0.1)	6 (0.3)	2 (0.1)	0 (0.0)
Employment	2627 (73.5)	1140 (92.0)	1027 (74.5)	460 (48.1)	693 (13.0)	372 (17.9)	257 (12.0)	64 (5.8)
Unemployment	284 (7.9)	69 (5.6)	115 (8.3)	100 (10.4)	4393 (82.3)	1697 (87.1)	1771 (82.4)	925 (21.1)
Retired	653 (18.3)	25 (2.0)	233 (16.9)	395 (41.3)	243 (4.6)	2 (0.1)	119 (5.5)	122 (11.0)
Smoking								
Non‐smoker	2075 (58.0)	725 (58.5)	752 (54.6)	598 (62.4)	4064 (76.1)	1654 (79.5)	1590 (74.0)	820 (73.8)
Ex‐smoker	538 (15.0)	150 (12.1)	221 (16.0)	167 (17.4)	331 (6.2)	80 (3.8)	137 (6.4)	114 (10.3)
Current smoker	962 (26.9)	364 (29.4)	405 (29.4)	193 (20.1)	945 (17.7)	346 (16.6)	422 (19.6)	177 (16.9)
BMI	26.34 ± 4.15	26.3 ± 4.36	26.45 ± 4.10	26.62 ± 3.89	28.91 ± 4.83	28.53 ± 4.86	29.18 ± 4.74	29.20 ± 4.91
Diabetes mellitus	479 (13.6)	87 (7.1)	207 (15.3)	185 (19.6)	769 (14.6)	162 (7.9)	335 (15.8)	272 (25.0)
Hypertension	1031 (28.9)	196 (15.8)	401 (29.1)	434 (45.4)	1712 (32.1)	308 (14.9)	797 (37.1)	607 (54.7)
Cardiovascular diseases	91 (2.5)	5 (0.4)	38 (2.8)	48 (5.5)	59 (1.1)	9 (0.4)	22 (1.0)	28 (2.5)

### 3.1 Prevalence of Minnesota codes related to cardiac ischemia

Detailed prevalence of Minnesota codes related to cardiac ischemia is indicated in Table 2. Based on Minnesota coding system, Q wave in precordial leads (5% in men vs. 3.2% in women), Q wave in aVL lead (1.4% in men vs. 1.2% in women), and Q wave in inferior leads (4.4% in men vs. 3.3% in women) were found at a higher prevalence. The most frequent abnormality in the Q wave in precordial leads was 1‐1‐2 (The Q wave in leads I or II or precordial leads, with a deviation of Q wave ≥0.04 s), and 1‐2‐4 (The Q wave in lead III with a deviation between 0.04 and 0.05 s with a Q wave with a height ≥1 mm in most of the aVF lead bits.) was also more frequent than other codes in the Q wave in inferior leads. In comparison between men and women the prevalence of Q wave in precordial leads (*p* < .001) and in inferior leads (*p* = .004) were significantly different. Furthermore, prevalence of Q wave in precordial leads was significantly different between age groups in men while prevalence of Q wave in aVL lead was significantly different in women between different age groups. The QS wave in precordial leads was found in 1.7% of men and 1.8% of women (*p* = .209) and significantly increased with age in women. Also, we found QS pattern in inferior leads was more common in men (3.4%) than women (2.0%; *p* < .001) which was significant increased with age in women. Focusing on the ST segment indicated that 1.3% of men and 2.0% of women in the population presented with major ST segment abnormalities which was significantly different (*p* = .003), while 0.4% of men and 0.8% of women were found to have minor ST segment abnormalities changes which also not significantly the same (*p* = .033). Major and minor ST segment frequencies was not different in between three age group. On the other hand, T‐wave analysis showed that 1.7% of men and 5.0% of women had major T‐wave abnormalities which was significantly different between men and women (*p* < .001) and the 5‐2 Minnesota code was higher in this category. Moreover, the prevalence of major T wave was significantly increased with age in both men and women. Minor T‐wave abnormalities was significantly more prevalent in men (2.0 in men vs. 1.0 in women; *p* < .001) which significantly increased with age only in women.

**TABLE 2:** Prevalence of Minnesota codes related to cardiac ischemia.

Categories^a^	Codes	Men (*N* = 3615)				Women (*N* = 5420)				*p*‐value*
		Total	35–44	45–54	55–65	Total	35–44	45–54	55–65	
Q wave in precordial leads	1‐1‐1	28 (0.8)	4 (0.3)	10 (0.7)	14 (1.4)	8 (0.1)	1 (0.0)	4 (0.2)	3 (0.3)	<.001^α^
	1‐1‐2	105 (2.9)	5 (2.0)	41 (3.0)	39 (4.1)	96 (1.8)	33 (1.6)	43 (2.0)	20 (1.8)	<.001^α^
	1‐2‐1	4 (0.1)	2 (0.2)	0	2 (0.2)	7 (0.1)	2 (0.1)	5 (0.2)	0	.530
	1‐2‐2	52 (1.5)	18 (1.3)	20 (1.5)	14 (1.5)	46 (0.9)	19 (0.9)	19 (0.9)	8 (0.7)	.006
	1‐3‐1	10 (0.3)	1 (0.1)	3 (0.2)	6 (0.6)	16 (0.3)	3 (0.1)	4 (0.2)	9 (0.8)	.517^α^, ^β^
	Total	199 (5.0)	30 (3.9)	74 (5.4)	75 (7.8)	173 (3.2)	58 (2.7)	75 (4.1)	40 (3.6)	<.001^α^
Q wave in aVL lead	1‐1‐3	21 (0.6)	5 (0.4)	10 (0.7)	6 (0.6)	34 (0.6)	10 (0.5)	11 (0.5)	13 (1.2)	.443^β^
	1‐3‐3	29 (0.8)	8 (0.6)	8 (0.6)	13 (1.4)	33 (0.6)	12 (0.6)	9 (0.4)	12 (1.1)	.172
	Total	50 (1.4)	13 (1.0)	18 (1.3)	19 (2.0)	67 (1.3)	22 (1.1)	20 (0.9)	25 (2.3)	.310^β^
Q wave in inferior leads	1‐1‐4	40 (1.1)	7 (0.6)	16 (1.2)	17 (1.8)	31 (0.6)	9 (0.4)	9 (0.4)	13 (1.2)	.004^α^, ^β^
	1‐1‐5	101 (0.3)	2 (0.2)	5 (0.4)	3 (0.3)	10 (0.2)	2 (0.1)	6 (0.3)	2 (0.2)	.247
	1‐2‐4	56 (1.6)	16 (1.3)	17 (1.2)	23 (2.4)	68 (1.3)	24 (1.2)	28 (1.3)	16 (1.4)	.143
	1‐2‐5	12 (0.3)	4 (0.3)	5 (0.4)	3 (0.3)	14 (0.3)	2 (0.1)	4 (0.2)	8 (0.7)	.330^β^
	1‐3‐4	20 (0.6)	9 (0.7)	7 (0.5)	4 (0.4)	26 (0.5)	12 (0.6)	12 (0.6)	2 (0.2)	.372
	1‐3‐5	20 (0.6)	10 (0.8)	7 (0.5)	3 (0.3)	24 (0.4)	6 (0.3)	13 (0.6)	5 (0.5)	.282
	Total	158 (4.4)	48 (3.9)	57 (4.2)	53 (5.5)	173 (3.3)	55 (3.7)	72 (3.4)	46 (4.2)	.004
QS wave in precordial leads	1‐1‐7	9 (0.3)	0	7 (0.5)	2 (0.2)	8 (0.1)	0	3 (0.1)	5 (0.5)	.201^α^, ^β^
	1‐2‐7	11 (0.3)	3 (0.2)	3 (0.2)	5 (0.5)	38 (0.7)	9 (0.4)	17 (0.8)	12 (1.1)	.007
	1‐3‐2	41 (1.1)	17 (1.4)	16 (1.2)	8 (0.8)	59 (1.1)	23 (1.1)	20 (0.9)	16 (1.4)	.465
	Total	61 (1.7)	20 (1.4)	26 (2.9)	15 (1.5)	105 (1.8)	32 (1.5)	40 (1.8)	32 (3.0)	.209^β^
QS pattern in inferior leads	1‐2‐3	27 (0.8)	9 (0.7)	7 (0.5)	11 (1.1)	19 (0.4)	5 (0.2)	7 (0.3)	7 (0.6)	.008
	1‐3‐6	82 (2.3)	24 (1.9)	30 (2.2)	28 (2.9)	72 (1.3)	17 (0.8)	35 (1.6)	20 (1.8)	.001^β^
	1‐3‐7	9 (0.3)	1 (0.1)	5 (0.4)	3 (0.3)	15 (0.3)	4 (0.2)	7 (0.3)	4 (0.4)	.485
	Total	118 (3.4)	34 (2.7)	42 (3.1)	42 (4.3)	106 (2.0)	26 (1.2)	49 (2.2)	31 (2.8)	<.001^β^
Major ST segment abnormalities	4‐1‐1	4 (0.1)	2 (0.2)	1 (0.1)	1 (0.1)	6 (0.1)	1 (0.0)	1 (0.0)	4 (0.4)	.631^β^
	4‐1‐2	21 (0.6)	4 (0.3)	8 (0.6)	9 (0.9)	55 (1.0)	16 (0.8)	22 (1.0)	17 (1.5)	.016
	4‐2	20 (0.6)	7 (0.6)	4 (0.3)	9 (0.9)	46 (0.9)	14 (0.7)	22 (1.0)	10 (0.9)	.065
	Total	45 (1.3)	13 (1.1)	13 (1.0)	19 (1.9 0	107 (2.0)	31 (1.5)	45 (2.0)	31 (2.8)	.003
Minor ST segment abnormalities	4‐3	3 (0.1)	0	1 (0.1	2 (0.2)	4 (0.1)	1 (0.0)	1 (0.0)	2 (0.2)	.582
	4‐4	12 (0.3)	6 (0.5)	3 (0.2	3 (0.3)	36 (0.7)	14 (0.7)	14 (0.7)	8 (0.7)	.021
	Total	15 (0.4)	6 (0.5)	4 (0.3)	5 (0.5)	40 (0.8)	15 (0.7)	15 (0.7)	10 (0.9)	.033
Major T‐wave abnormalities	5‐1	11 (0.3)	1 (0.1)	3 (0.2)	7 (0.7)	14 (0.3)	4 (0.2)	7 (0.3)	3 (0.3)	.418^α^
	5‐2	87 (2.4)	18 (1.5)	35 (2.5)	34 (3.5)	253 (4.7)	72 (3.5)	112 (5.2)	69 (6.2)	<.001^α^, ^β^
	Total	98 (2.7)	19 (1.6)	38 (2.7)	41 (4.2)	267 (5.0)	76 (3.7)	119 (5.5)	72 (6.5)	<.001^α^, ^β^
Minor T‐wave abnormalities	5‐3	58 (1.6)	15 (1.2)	23 (1.7)	20 (2.1)	160 (0.3)	50 (2.4)	57 (2.7)	53 (4.8)	<.001^β^
	5‐4	16 (0.4)	7 (0.6)	5 (0.4)	4 (0.4)	37 (0.7)	14 (0.7)	17 (0.8)	6 (0.5)	.089
	Total	74 (2.0)	22 (1.8)	28 (2.1)	24 (2.5)	197 (1.0)	64 (3.1)	74 (3.5)	59 (5.3)	<.001^β^

* *p* value between men and women.

^α^ *p* value <.05 between age groups in men.

^β^ *p* value <.05 between age groups in women.

^a^ Expressed in *n* (%).

### 3.2 Prevalence of Minnesota codes related to conduction defects

According to the reported ECGs, prevalence of atrial block (first degree) was 0.2% in men versus 0.3% in women. Wolff‐Parkinson‐White Pattern (WPW) was found in 0.2% of women which significantly higher in compare with men (*p* = .019. Complete LBBB and RBBB was found at a higher frequency in men which was not significant, while they were significantly more prevalent among older women. The R‐R′ pattern was found at a higher frequency in men too (0.7% in men vs. 0.5% in women) which was not significant (*p* = .262). LAH in our study was found more prevalent in men (0.3% in men vs. 0.2% in women) which was not significant. Brugada pattern had a higher prevalence in men than women (*p* = .001), while there was no relation was found with age. In the end, some of the conduction defects like A‐V block (second degree), Wenckebach's Phenomenon, intermittent LBBB, incomplete RBBB, and intra‐ventricular block did not have a significant frequency (Table 3).

**TABLE 3:** Prevalence of Minnesota codes related to conduction defects.

Categories^a^	Codes	Men (*N* = 3615)				Women (*N* = 5420)				*p* value*
		Total	35–44	45–54	55–65	Total	35–44	45–54	55–65	
A‐V block (2nd degree)	6‐1	0	0	0	0	0	0	0	0	–
	6‐2‐1	1 (0.0)	1 (0.1)	0	0	1 (0.0)	0	1 (0.1)	0	.641
	6‐2‐2	0	0	0	0	0	0	0	0	–
Wenckebach's Phenomenon	6‐2‐3		0	0	0	1 (0.0)	1 (0.0)	0	0	.599
AB (1st degree)	6‐3	7 (0.2)	1 (0.1)	3 (0.2)	3 (0.3)	16 (0.3)	5 (0.2)	5 (0.2)	6 (0.5)	.234
WPW	6‐4‐1		0	0	0		0	0	0	–
	6‐4‐2		0	0	0		0	0	0	–
	6‐5	1 (0.0)	1 (0.1)	0	0	11 (0.2)	5 (0.2)	3 (0.1)	3 (0.3)	.019
Complete LBBB	7‐1‐1	20 (0.6)	6 (0.5)	8 (0.6)	6 (0.6)	25 (0.5)	7 (0.3)	4 (0.2)	14 (1.3)	.326β
Intermittent LBBB	7‐1‐2	0 (0.0)	0	0	0	2 (0.0)	1 (0.0)	0	1 (0.1)	.359
Complete RBBB	7‐2‐1	15 (0.4)	2 (0.2)	6 (0.4)	7 (0.7)	11 (0.2)	1 (0.0)	4 (0.2)	6 (0.5)	.053β
Incomplete RBBB	7‐3	4 (0.1)	2 (0.2)	1 (0.1)	1 (0.1)	4 (0.1)	1 (0.0)	2 (0.1)	1 (0.1)	.408
Intra‐ventricular block	7‐4	1 (0.0)	1 (0.1)	0	0	2 (0.0)	0	2 (0.1)	0	.647
R‐R′ pattern	7‐5	24 (0.7)	11 (0.9)	7 (0.5)	6 (0.6)	29 (0.5)	14 (0.7)	9 (0.4)	6 (0.5)	.262
Incomplete LBBB	7‐6	6 (0.2)	2 (0.2)	1 (0.1)	3 (0.3)	4 (0.1)	1 (0.0)	2 (0.1)	1 (0.1)	.168
LAH	7‐7	10 (0.3)	2 (0.2)	3 (0.2)	5 (0.5)	10 (0.2)	3 0.1	4 (0.2)	3 (0.3)	.247
Brugada pattern	7‐9‐1	0 (0.0)	0	0	0	1 (0.0)	1 (0.0)	0	0	.599
	7‐9‐2	12 (0.3)	7 (0.6)	5 (0.4)	0	4 (0.1)	0	2 (0.1)	2 (0.2)	.005
	7‐9‐3	8 (0.2)	3 (0.2)	4 (0.3)	1 (0.1)	2 (0.0)	1 (0.0)	1 (0.0)	0	.012
	Total	20 (0.5)	10 (0.8)	9 (0.7)	1 (0.1)	7 (0.2)	2 (0.1)	3 (0.2)	2 (0.2)	.001
Fragmented QRS	7‐10	67 (1.9)	27 (2.2)	23 (1.7)	17 (1.8)	79 (1.5)	24 (1.2)	38 (1.8)	17 (1.5)	.088

Abbreviations: AB, Atrial block; LAH, Left anterior hemiblock; LBBB, left bundle branch block; RBBB, right bundle branch block; WP, Wenckebach's Phenomenon; WPW, Wolff‐Parkinson‐White Pattern.

* *p* value between men and women.

^α^ *p* value <.05 between age groups in men.

^β^ *p* value <.05 between age groups in women.

^a^ Expressed in *n* (%).

### 3.3 Prevalence of Minnesota codes related to arrhythmias

According to the Minnesota coding system, the most common arrhythmia in the study population was sinus bradycardia (heart rate less than 50/min) and it was higher in men (4.8%) than in women (1.4%; *p* < .001) and more prevalent in older age group only in women, while sinus tachycardia (heart rate over 100/min) and premature ventricular contractions (PVCs) were the most prevalent after sinus bradycardia. Sinus tachycardia was found more in women (1.9%) than in men (1.1%) opposite to bradycardia (*p* = .001). PVCs prevalent was (1.2%) in both gender and increased with age in males. Also, PACs almost had the same prevalence in both genders, but it was significantly higher in the older age group of women (55–65). Sinoatrial arrest and sinoatrial block were found in only 0.1% of the population of any gender. Eventually, other arrhythmias like wandering pacemaker, premature beats, persistent ventricular (idioventricular) rhythm, intermittent ventricular tachycardia, ventricular parasystole, supraventricular rhythm persistent, atrial fibrillation, atrial flutter, intermittent atrial fibrillation, intermittent atrial flutter, and other arrhythmias were not found in the population sample. (Table 4).

**TABLE 4:** Prevalence of Minnesota codes related to arrhythmias.

Categories^a^	Codes	Men (*N* = 3615)				Women (*N* = 5420)				*p* value*
		Total	35–44	45–54	55–65	Total	35–44	45–54	55–65	
PAC	8‐1‐1	8 (0.2)	3 (0.2)	3 (0.2)	2 0.2	17 (0.3)	3 (0.1)	4 (0.2)	10 (0.9)	.270β
PVC	8‐1‐2	42 (1.2)	9 (0.7)	19 (1.4)	14 (1.5)	62 (1.2)	21 (1.0)	20 (0.9)	21 (1.9)	.514β
Wandering pacemaker	8‐1‐4	0 (0.0)	0	0	0	1 (0.0)	1 (0.0)	0	0	.599
Premature beats	8‐1‐5	0 (0.0)	0	0	0	1 (0.0)	0	0	1 (0.1)	.599
Persistent ventricular (idioventricular) rhythm	8‐2‐2	1 (0.0)	0	1 0.1	0	0 (0.0)	0	0	0	.401
Intermittent VT	8‐2‐3	0 (0.0)	0	0	0	1 (0.0)	1 (0.0)	0	0	.599
Ventricular parasystole	8‐2‐4	0 (0.0)	0	0	0	0 (0.0)	0	0	0	.401
SV rhythm persistent	8‐4‐1	1 (0.0)	0	1 (0.1)	0	2 (0.0)	2 (0.1)	0	0	.647
AF	8‐3‐1	2 (0.1)	0	1 (0.1)	1 (0.1)	2 (0.0)	0	0	2 (0.2)	.527β
AFL	8‐3‐2	0 (0.0)	0	0	0	0 (0.0)	0	0	0	–
Intermittent AF	8‐3‐3	0 (0.0)	0	0	0	1 (0.0)	1 0.0	0	0	.599
Intermittent AFL	8‐3‐4	0 (0.0)	0	0	0	0 (0.0)	0	0	0	–
Sinoatrial arrest	8‐5‐1	2 (0.1)	0	1 (0.1)	1 (0.1)	3 (0.1)	1 (0.0)	2 (0.1)	0	.681
Sinoatrial block	8‐5‐2	3 (0.1)	1 (0.1)	1 (0.1)	1 (0.1)	5 (0.1)	3 (0.1)	1 (0.0)	1 (0.1)	.592
Sinus tachycardia	8‐7	38 (1.1)	13 1.0	13 (0.9)	12 (1.3)	101 (1.9)	30 (1.4)	50 (2.3)	21 (1.9)	.001
Sinus bradycardia	8‐8	171 (4.8)	57 (4.6)	64 (4.6)	50 (5.2)	75 (1.4)	16 (0.8)	37 (1.7)	22 (2.0)	<.001β
Other arrhythmias	8‐9	0 (0.0)	0	0	0	4 (0.1)	1 (0.0)	3 (0.1)	0	.129

Abbreviations: AF, Atrial fibrillation; AFL, Atrial flutter; PAC, premature atrial complex; PVC, premature ventricular complex; SV, Supraventricular; VT, Ventricular tachycardia.

* *p* value between men and women.

^α^ *p* value <.05 between age groups in men.

^β^ *p* value <.05 between age groups in women.

^a^ Expressed in *n* (%).

### 3.4 Prevalence of other Minnesota codes

QRS axis deviation analysis of the participants' ECGs showed that the left QRS axis deviation was more prevalent than other axis deviations and also it was higher in men (5.1% in men vs. 3.0% in women; *p* < .001), and it increased with age which was statistically significant only in women. Other QRS axis deviations were found at similar frequencies in both genders. Also, there is a significant relationship with age regard to right axis deviation (code 2‐2) only in men. Further analyses of the recorded ECGs determined that LVH was higher in men (1.1% in men vs. 0.5% in women; *p* = .003), as well as 3‐1 code was found more than others. RVH and high P‐wave amplitude prevalence were the same in both genders. ST segment elevation was found high prevalent in our study population (4.5%). It was almost four times higher in men than in women (9.1% vs. 2.0%; *p* < .001) and more frequent in younger men. Beside previous abnormalities, low QRS amplitude was found higher in women (1.6% in men vs. 1.9% in women) which was not significant (*p* = .203). High T‐wave amplitude was significantly higher in men (1.8% in men vs. 1.0% in women; *p* < .001). Due to the results notched and widened P wave in men was (2.9%) and in women was (1.9%; *p* = .001) and as well, it was significantly related to age in both gender and increased with age in women. At last, definite early repolarization and probable early repolarization were also higher in men than women in the population (accordingly, *p* < .001 and *p* = .016; Table 5).

**TABLE 5:** Prevalence of other Minnesota codes.

Categories^a^	Codes	Men (*N* = 3615)				Women (*N* = 5420)				*p* value*
		Total	35–44	45–54	55–65	Total	35–44	45–54	55–65	
QRS axis deviation	2‐1	183 (5.1)	53 (4.3)	70 (5.1)	60 (6.3)	160 (3.0)	42 (2.0)	64 (3.0)	54 (4.9)	<.001^β^
	2‐2	12 (0.3)	0	5 (0.4)	7 (0.7)	13 (0.2)	3 (0.1)	7 (0.3)	3 (0.3)	.271α
	2‐3	15 (0.4)	6 (0.5)	4 (0.3)	5 (0.5)	26 (0.5)	11 0.5	9 0.4	6 (0.5)	.386
	2‐4	2 (0.1)	1 (0.1)	1 (0.1)	0	4 (0.1)	2 (0.1)	1 (0.1)	1 (0.1)	.542
	2‐5	6 (0.2)	1 (0.1)	3 (0.2)	2 (0.2)	9 (0.2)	4 (0.2)	1 (0.0)	4 (0.4)	.607
LVH	3‐1	28 (0.8)	8 (0.6)	13 (0.9)	7 (0.7)	17 (0.3)	6 (0.3)	5 (0.2)	6 (0.5)	.002
	3‐3	10 (0.3)	4 (0.3)	6 (0.4)	0	11 (0.2)	2 (0.1)	6 (0.3)	3 (0.3	.312
	Total	38 (1.1)	12 (0.9)	19 (1.3)	7 (0.7)	28 (0.5)	8 (0.4)	11 (0.5)	9 (0.8)	.003
RVH	3‐2	3 (0.1)	1 (0.1)	2 (0.1)	0	8 (0.1)	3 (0.1)	5 (0.2)	0	.294
Low QRS amplitude	9‐1	57 (1.6)	13 (1.0)	26 (1.9)	18 (1.9)	99 (1.9)	38 (1.8)	39 (1.8)	22 (2.0)	.203
STE	9‐2	324 (9.1)	139 (11.2)	127 (9.2)	58 (6.1)	109 (2.0)	43 (2.1)	43 (2.0)	23 (2.1)	<.001^α^
Tall P wave	9‐3	15 (0.4)	1 (0.1)	9 (0.7)	5 (0.5)	22 (0.4)	8 (0.4)	11 (0.5)	3 (0.3)	.541
Tall T wave	9‐5	64 (1.8)	30 (2.4)	24 (1.7)	10 (1.0)	4 (0.1)	2 (0.0)	2 (0.1)	1 (0.1)	<.001
Notched and widened P wave	9‐6	105 (2.9)	19 (1.5)	58 (4.2)	28 (2.9)	10 (1.9)	26 (1.3)	35 (1.6)	40 (3.6)	.001^α^, ^β^
Definite ER	9‐7‐1	30 (0.8)	13 (1.0)	10 (0.7)	7 (0.7)	16 (0.3)	10 (0.5)	4 (0.2)	2 (0.2)	<.001
Probable ER	9‐7‐2	28 (0.8)	5 (0.4)	13 (0.9)	10 (1.0)	22 (0.4)	3 (0.1)	13 (0.6	6 (0.5)	.016^β^

Abbreviations: ER, early repolarization; LVH, left ventricular hypertrophy; RVH, right ventricular hypertrophy; STE, ST segment elevation; Tall P wave, P‐wave amplitude ≥2.5 mm; Tall T wave, T‐wave amplitude >12 mm.

^a^ Expressed in *n* (%).

* *p* value between men and women.

^α^ *p* value <.05 between age groups in men.

^β^ *p* value <.05 between age groups in women.

### 3.5 Comparison of the heart rate and ECG duration in gender and age groups

**FIGURE 1:**
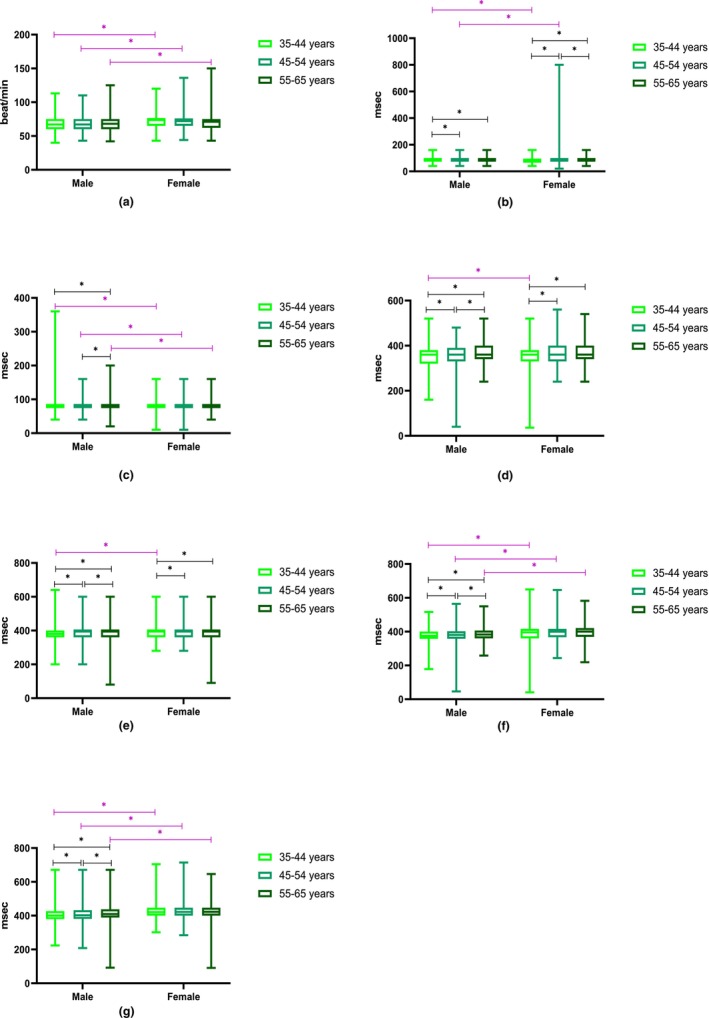
Differences of heart rate, QRS duration, Max QT, Min QT, Max QTc, Min QTc, and P‐wave duration in different groups of age and sex.

Comparison of the heart rate, P‐wave duration, and QRS duration between men and women indicated that there were significant differences between them (*p* < .001); while there were no significant differences between men and women in terms of minimum QT duration (*p* = .067) and maximum QT duration (*p* = .378). Despite there were significant differences in the P wave, minimum QT, and maximum QT duration between different age groups of male (*p* < .001), females presented significant differences in their heart rate (*p* = .720), P‐wave duration (*p* < .001), QRS duration (*p* = .002), minimum QT (*p* < .001), and maximum QT duration (*p* = .001) between categorized age groups. Moreover, minimum QTc and maximum QTc analysis revealed significant differences in both males and females due to their age categorization (*p* < .001; Table 6; Figure 1).

**TABLE 6:** Comparison of heart rate, wave, and segment duration in different groups.

Variables	Male				*p*‐value^1^	Female				*p*‐value^2^	*p*‐value*
	Total	35–44	45–54	55–65		Total	35–44	45–54	55–65		
Heart rate (beat/min)	67.99 ± 10.77	67.93 ± 10.66	67.91 ± 10.61	68.27 ± 11.17	.695	72.24 ± 10.82	72.52 ± 10.23	72.30 ± 11.03	71.61 ± 11.41	.720	<.001
P‐wave duration (ms)	87.07 ± 16.28	85.70 ± 15.67	87.30 ± 16.36	88.60 ± 17.01	<.001^α,β^	85.36 ± 18.06	83.80 ± 14.72	85.7 ± 21.62	87.3 ± 16.46	<.001^α,β,μ^	<.001
QRS duration (ms)	81.37 ± 14.72	81.50 ± 15.73	80.80 ± 13.72	81.80 ± 14.74	.256	79.17 ± 13.53	78.6 ± 13.19	79.10 ± 13.28	80.40 ± 14.74	.003^β,μ^	<.001
Min QT (ms)	359.06 ± 35.30	355.00 ± 34.48	359.93 ± 35.79	363.8 ± 35.15	<.001 ^α,β,μ^	360.45 ± 34.90	358.10 ± 35.01	361.10 ± 34.36	363.80 ± 35.56	<.001^α,β^	.067
Max QT (ms)	385.51 ± 38.73	380.60 ± 41.15	385.20 ± 42.50	388.70 ± 45.09	<.001α ^,^ β ^,^ μ	386.53 ± 34.46	383.00 ± 37.01	386.40 ± 38.33	388.10 ± 48.19	.001^α,β^	.204
Min QTc (ms)	380.08 ± 38.83	375.64 ± 37.56	380.42 ± 40.30	385.4 ± 37.59	<.001α ^,^ β ^,^ μ	393.40 ± 39.62	391.79 ± 39.74	394.14 ± 39.56	395.14 ± 39.62	.044	<.001
Max QTc (ms)	407.24 ± 47.34	402.82 ± 45.42	407.68 ± 47.77	412.06 ± 49.10	<.001α ^,^ β ^,^ μ	420.81 ± 46.00	419.67 ± 42.71	421.94 ± 45.07	421.62 ± 53.72	.040	<.001

* Between male and female.

^1^ Between age groups in male.

^2^ Between age groups on female.

^α^ *p* value <.05 between age group of 35–44 and 45–54.

^β^ *p* value <.05 between age group of 35–44 and 55–65.

^μ^ *p* value <.05 between age group of 45–54 and 55–65.

## 4 DISCUSSION

A 12‐lead ECG may be a reliable way for the detection, prediction, and prevention of cardiovascular events, large epidemiological studies are needed to identify the ECG abnormalities in the general population to improve the accuracy of the ECG interpretation methods. Moreover, there have been few reports from different populations concerning the prevalence of abnormal findings in resting ECGs evaluated by the Minnesota Coding system. Therefore, we analyzed the prevalence of ECG abnormalities in the adult subjects of the MASHAD study, who were recruited from the general population of 35–65 years old of Mashhad, Iran. Our primary findings determined that nearly half of the studied population have presented at least one abnormality in their resting 12‐lead ECGs. Generally, women presented higher frequencies of ECG abnormalities than men, except at the ages between 35 to 44 years old.

In our studied population, we observed that Q wave and QS pattern abnormalities comprised the highest prevalence of ECG abnormalities. The frequency of these patterns was a little higher in women than in men. Studies by Piwońska et al. (2019) and Prineas et al. (2012) in Poland and the United States, respectively, indicated that nearly one third and one quarter of the Polish and American population presented Q wave and QS pattern abnormalities in their resting ECGs (women more frequently); while these phenomena were too less frequent (approximately 1%) in Chines population that investigated by Yu et al. (2020). In addition, even minor Q waves on an ECG could be associated with death or hospitalization, regardless of age, hypertension, or diabetes (Godsk et al., 2012). However, the high prevalence of Q‐wave and QS‐pattern abnormalities in our studied population could be due to race, age, BMI, and underlying conditions such as a history of diabetes, hypertension, and CVDs. Additionally, lifestyle and dietary habits which affect lipid profile could play a role in this situation.

About 5% of our studied population presented any axis deviation; 80% of them were left axis deviation Previous studies in the Chinese and Indian populations showed a similar frequency of axis deviation (4% and 5%, respectively; Krishnan et al., 2022; Yu et al., 2020). Ventricular hypertrophy was not common in our population, approximately 5% of the Chinese population and less than 1% of Brazilian study presented either left or right ventricular hypertrophy (Yu et al., 2020); which can commonly observe due to long‐time hypertension (Pinto‐Filho et al., 2017; Yildiz et al., 2020). Additionally, several factors could be the reason for these differences such as the higher mean age of our study, race, being under the treatment of hypertension, previous history of diabetes and CVDs, and type of medications. If fact, hypertension is a common reason for hypertension, but good control of blood pressure can explain less prevalence of hypertrophy in the population.

More than 3% of our study population presented either major or minor ST segment depression which was more frequently in females. Moreover, the prevalence of T‐wave inversion was two times higher in our studied population than ST segment inversion. It should be noted that both ST segment depression and T‐wave inversion can be observed in ischemic events. In an Indian study by Khane and Surdi it have been found that the prevalence of ST segment and/or T‐wave abnormalities were observed more in women similar to our study population (Khane & Surdi, 2012); opposite to our study repolarization abnormalities such as ST segment depression and negative T wave was more prevalent in males than females in the Dutch population (van der Ende et al., 2017).

According to recent data the atrioventricular block is considered the most prevalent cause of idiopathic fibrosis and sclerosis of the conduction system (Kashou et al., 2021); also short P‐R interval may be related to pre‐excitation syndromes and junctional arrhythmia (Staikou et al., 2018). In our study, approximately 0.4% of individuals involved A‐V conduction defects which were almost composed of first‐degree A‐V block and short P‐R interval; in comparison to other studies, our findings showed lower frequencies than Polish, Indian and Thai populations (Krishnan et al., 2022; Piwońska et al., 2019; Sriratanaviriyakul et al., 2010). In all mentioned populations A‐V conduction defects were more in females, like in our study, but in American Indians, the frequency of this abnormality was higher in males (Oopik et al., 1996).

Ventricular conduction, 3.7% of individuals showed these kinds of abnormalities. Left bundle branch block (LBBB), right bundle branch block (RBBB), and R‐R′ in V_1_ or V_2_ precordial lead were more prevalent. Fragmented QRS was reported in 1.6% of our subjects. It should be mentioned that males approximately had equal frequency in different ages; however, 55–65‐year females had a higher prevalence of LBBB. The prevalence of LBBB was the same in both genders eventually, although in Dutch, Indian, and Chinese people LBBB was significantly higher in women (Krishnan et al., 2022; van der Ende et al., 2017; Yu et al., 2020). Conversely, Khane et al found it more prevalent in Indian males (Khane & Surdi, 2012). RBBB was more prevalent in males and can be a sign of pulmonary embolism, right‐sided heart failure, cardiomyopathy, and myocarditis (Harkness & Hicks, 2021). Despite the Polish study, R‐R′ in V_1_ or V_2_ precordial lead did not show any significant gender difference in our study (Piwońska et al., 2019). Also, the left anterior fascicular block was more common in males similar to the Indian population (Krishnan et al., 2022); on the contrary, LAH was reported only in males in the polish study (Piwońska et al., 2019). Note that LAH can lead patients to congestive heart failure, atrial fibrillation, and death (Ali et al., 2015).

Brugada patterns, characterized by specific electrocardiographic abnormalities, have garnered significant attention in the field of cardiology due to their association with an increased risk of sudden cardiac death (SCD; Sieira & Brugada, 2017). These distinctive patterns, typically observed in the right precordial leads (V_1_–V_3_), are characterized by ST segment elevation followed by a negative T wave, often resembling a coved or saddleback configuration. Although initially considered benign, further research has revealed their potential as a marker for life‐threatening ventricular arrhythmias (Coppola et al., 2021). The underlying pathophysiological mechanisms of Brugada patterns remain incompletely understood; however, genetic mutations affecting sodium channel function have been implicated in the majority of cases (Li et al., 2020). Diagnosis of Brugada syndrome is crucial, as it enables risk stratification and implementation of appropriate therapeutic strategies, including implantable cardioverter‐defibrillator placement. Additionally, the identification of Brugada patterns in patients without overt symptoms or a family history of SCD remains a challenge, emphasizing the need for improved screening and diagnostic tools (Pappone & Santinelli, 2019). Our results revealed that the Brugada pattern had a higher prevalence in men than women, while there was no relation was found with age. However, investigations in Spain and the United States represented a lower rate of Brugada patterns in the studied populations (Patel et al., 2009; Rodríguez‐Capitán et al., 2016). These differences could be due to the studied population's age, race, and underlying conditions.

Arrhythmia was observed in 5.8% of the population; PVCs, tachycardia, and bradycardia being the most common types. We investigated that PVCs were more prevalent in 55–65‐year‐oldwomen while previous studies reported opposite result or same prevalence in different gender, also they showed it was age‐dependent (Piwońska et al., 2019; van der Ende et al., 2017). Most people may feel it as a result of stress, using caffeine or alcohol, and feeling their heartbeat skip. PVCs can increase the risk of cardiomyopathy in people (Piwońska et al., 2019). Similar to previous studies (Indian, Dutch, and Chinese population studies) tachycardia was more common in women and bradycardia was higher in males (Krishnan et al., 2022; van der Ende et al., 2017; Yu et al., 2020); in both of these arrhythmias, frequency got increased with age. If tachycardia is left untreated can lead to stroke, heart failure SCD (Gopinathannair et al., 2009).

Despite the frequency of abnormal ECG presentations being increased by aging in both genders, ST segment elevations were the most frequent finding in the studied population which was more frequent in males than females; however, more frequent high‐amplitude R waves, QRS axis deviation, arrhythmias in males, and repolarizing changes such as ST depressions and negative T waves in females were observed in the study form Poland by Piwońska et al. (2019). On the opposite side of our study, a study by Yu et al. in China reported the prevalence of the ST segment elevation as less than 1%; and they reported ST depression and T abnormalities as the most frequent ECG abnormalities in their studied Chinese population (Yu et al., 2020).

Low‐voltage QRS found in 1.7% of ECGs and was similar in both genders but in Dutch and south India it was significantly higher in females (Krishnan et al., 2022; van der Ende et al., 2017). ST elevation was four times higher in men in our study (totally, 4.5% in the population) similar to Dutch and Indian reports (Krishnan et al., 2022; van der Ende et al., 2017); but significantly higher in men in South India (Krishnan et al., 2022). ST elevation can be a sign of ventricles dying during heart attack. Early repolarization was also more prevalent in men. Unlike the past, recent studies demonstrated that it can be related to SCD and the dangers arrhythmia (Ali et al., 2015). Left atrial enlargement was higher in men at all, but it was significantly more frequent in the 45–55 men and 55–65 women age group. We demonstrated tall P‐wave amplitude same frequency in both genders and can result in right atrial enlargement due to pulmonary hypertension (Harrigan & Jones, 2002). Also, the tall T‐wave amplitude was found more prevalent in young men which can be an early sign of ST elevation myocardial infarction (Smith, 2005).

These differences between European, Asian, American, and Middle East populations could be due to their life‐style and nutritional habits which make middle‐eastern people at a higher risk of CVDs, especially myocardial infarction; however, genetic factors can have specific effects on these events.

Despite the large population evaluated in this study, it was conducted in a single city in Iran, which could be its major limitation; however, the present study was the first large population‐based study conducted in Iran. Additionally, our study was limited to the lack of the participants' follow‐up outcomes and some recorded characteristics of the participants such as race. Moreover, we were unable to report the participants' medications in the current stage of the study, which would be completed in future reports. Furthermore, further studies with larger populations and wider age ranges can cover the gaps of the present study. In most of the similar studies, the factors related to heart abnormalities, including obesity, hypertension, smoking, hyperlipidemia, and diabetes, were also considered along with the general problem, which in future discussions can lead to a better understanding of the ultimately CVDs.

## 5 CONCLUSION

The present study was conducted on a large population of one of the largest cities in Iran. Despite our findings indicating the middle level of ECG abnormalities in the studied population, it seems that the current level of abnormalities could be decreased by effective interventions. However, further studies are required to approve our findings and make them complete.

## AUTHOR CONTRIBUTIONS

All authors have read and approved the manuscript. Study concept and design: MG and MM; data collection: SSS, ME, HRR, HA, BS, AHB, FM, MV and MA; Analysis and interpretation of data: SSS, HE, NT, ML, MMS and RAD; Drafting of the manuscript: SSS, AMG MJ and FA; Critical revision of the manuscript for important intellectual content: GAF, MG and HRR.

## CONFLICT OF INTEREST STATEMENT

There is no conflict of interest.

## FUNDING INFORMATION

This study was funded by Mashhad University of Medical Sciences.

## ETHICS STATEMENT

The study protocol was given approval by the Ethics Committee of Mashhad University of Medical Sciences (IR.MUMS.MEDICAL.REC.1399.783) and written informed consent was obtained from participants.

## Data Availability

Data will be available upon request.
